# Supplementary material for: Ex Vivo Analysis of the Association of GFP-Expressing L. aethiopica and L. mexicana with Human Peripheral Blood-Derived (PBD) Leukocytes over 24 Hours
Source: Microorganisms. 2024 Sep 19;12(9):1909. doi: 10.3390/microorganisms12091909 (PMC11434358; doi:10.3390/microorganisms12091909)
Supplement: Supplementary file 1 [file microorganisms-12-01909-s001.zip › microorganisms-3175568-supplementary.pdf]

Supplementary Materials:

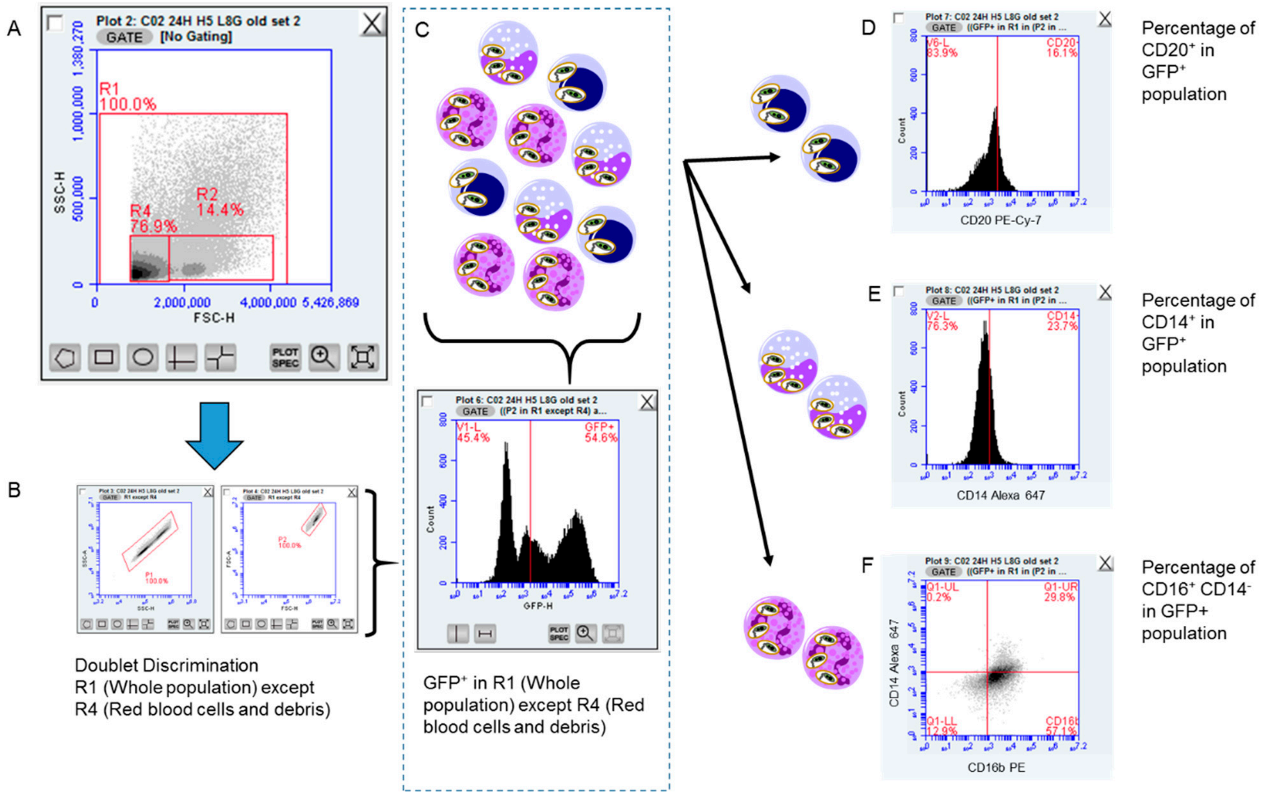

Figure S1. Gating strategy for whole blood cocktail 1 to separate neutrophils, monocytes and B cells within GFP positive populations. (A) The population of interest was selected following the gating of viable cells in the FSC-H vs SSC-H plot (R1). (B) Removal of cell doublets (SSC-H Vs SSC-A & FSC-H Vs FSC-H). (C) GFP positive population in R1 population. (D-F) Subpopulations of $CD20^+$ (B-lymphocytes), $CD14^+$ (Monocytes) and $CD16b^+CD14^-$ (Neutrophils) populations within GFP^+ population.

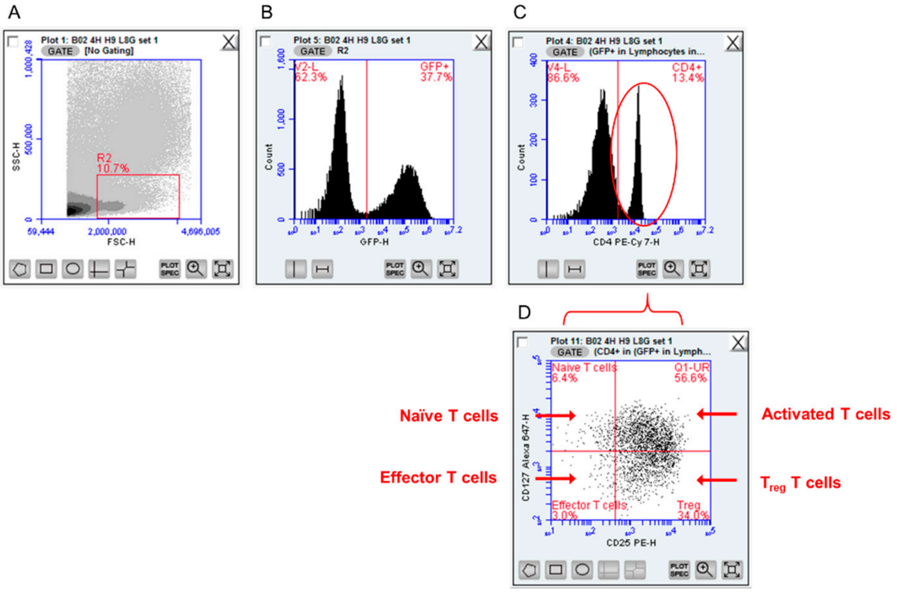

Figure S2. Gating strategy for whole blood cocktail 2 to separate subpopulations of T cells in GFP positive populations. (A) The population of lymphocytes was selected following the gating of viable cells in the FSC-H vs SSC-H plot (R1). (B) GFP positive population in R2 population. (C) CD4+ population within GFP+ population. (D) Subpopulations of $CD4^+$, $CD25^-$, $CD127^+$ (Naïve T cells), $CD4^+$, $CD25^+$, $CD127^+$ (Activated T cells), $CD4^+$, $CD127^-$, $CD25^-$ (Effector T-cells) and $CD4^+$, $CD25^+$, $CD127^-$ (Treg) populations within GFP^+ population.
